# Recent advances in understanding phosphoinositide signaling in the nervous system

**DOI:** 10.12688/f1000research.16679.1

**Published:** 2019-03-12

**Authors:** Eamonn James Dickson

**Affiliations:** 1 Department Physiology and Membrane Biology, University of California, Davis, CA, 95616, USA

**Keywords:** Endoplasmic reticulum, Voltage gated Ca2+ channel, Voltage gated K+ channel, Membrane contact site, Phosphatidylinositol, Phospholipase C, Polyphosphoinositide, Phospholipids, Plasma membrane, Phosphoinositide, Neuron, Ion channel

## Abstract

Polyphosphoinositides (PPIn) are essential signaling phospholipids that make remarkable contributions to the identity of all cellular membranes and signaling cascades in mammalian cells. They exert regulatory control over membrane homeostasis via selective interactions with cellular proteins at the membrane–cytoplasm interface. This review article briefly summarizes our current understanding of the key roles that PPIn play in orchestrating and regulating crucial electrical and chemical signaling events in mammalian neurons and the significant neuro-pathophysiological conditions that arise following alterations in their metabolism.

## Introduction

**Figure 1.**
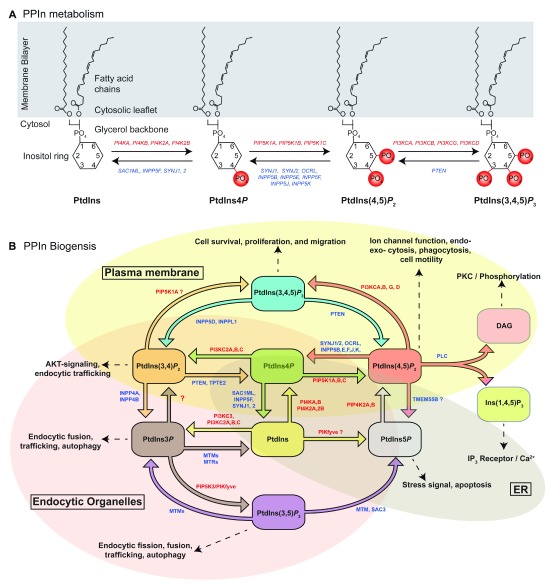
Phosphoinositide metabolism and biogenesis. ( **A**) Phosphoinositide metabolism. Hypothetical equilibrium reaction involving four polyphosphoinositide (PPIn) species at the membrane–cytosol interface. The basic structure of the parent PPIn, phosphatidylinositol (PtdIns), forms the substrate for subsequent PPIn species. Red labels represent gene names of lipid kinases that catalyze the addition of phosphate groups (phosphorylate) at specific positions of the inositol ring. Blue labels represent gene names of lipid phosphatases that remove phosphate groups (dephosphorylate) at specific positions of the inositol ring. ( **B**) Phosphoinositide biogenesis. Diagram summarizing the major PPIn lipid kinase and phosphatase reaction pathways. Red and blue labels are the gene names of enzymes capable of catalyzing each reaction. Gene names with question marks (?) represent enzymes with some uncertainty surrounding their ability to catalyze a specific reaction. Dashed arrows represent the major cellular roles for each individual PPIn. Colored circles represent the approximate cellular locations of each PPIn species. ER, endoplasmic reticulum; PKC, protein kinase C; PTEN, phosphatase and tensin homolog.

Polyphosphoinositides (PPIn) are a family of minor (low-abundance), negatively charged phospholipid molecules found on the cytoplasmic leaflet of all cellular membranes that play critical roles in membrane homeostasis and cellular signaling ^1^. Structurally, they consist of two fatty acid chains (that insert into the cytosolic leaflet of cellular membranes), a glycerol moiety, and an inositol headgroup ( Figure 1A).

In primary mammalian cells, about 80% of the phosphoinositide (PI) molecules have stearoyl/arachidonyl as their fatty acid chains ^2– 4^ ( Figure 1A, “Fatty acid chains”). Typically, this is designated C18:0/C20:4 (the number of carbons:number of double bonds in each fatty acid) or 38:4 for the whole molecule. A small but increasing body of evidence suggests that the fatty acid chains of a given PPIn themselves could represent a signaling code. For example, it has been suggested that different fatty acid chains may confer substrate preferences at the level of one or more lipid kinases and lipid phosphatases ^5, 6^; however, this is an area of work that requires further investigation.

**Figure 2.**
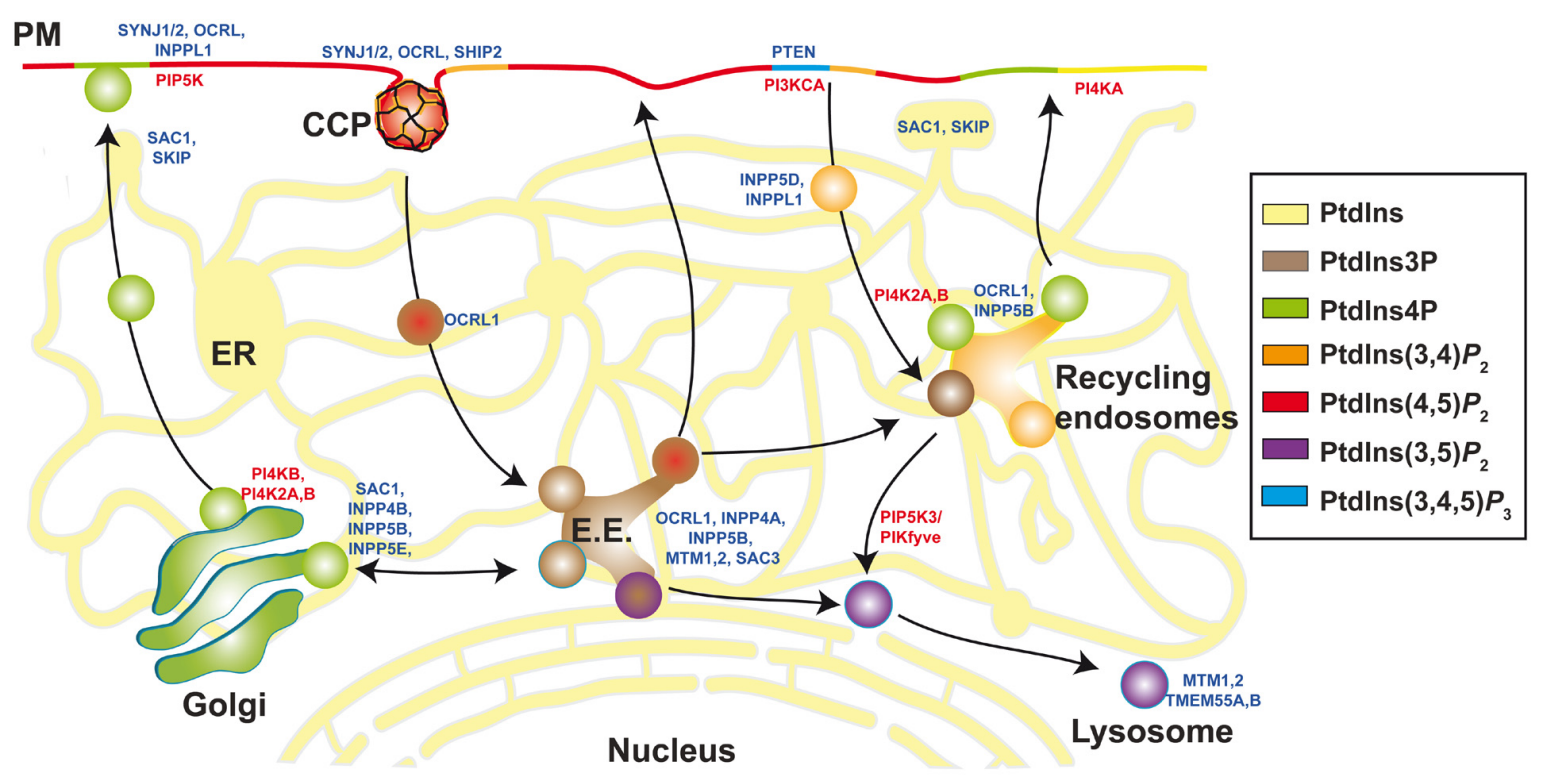
Phosphoinositide zip code. Cellular distribution of polyphosphoinositide (PPIn) species and metabolizing enzymes. Diagram depicting the signature distribution of each PPIn species and approximate location of enzymes regulating each species. Blue and red labels represent PPIn phosphatases and PPIn kinases, respectively. E.E., early endosome; ER, endoplasmic reticulum; PM, plasma membrane; PtdIns, phosphatidylinositol.

The vast majority of work detailing the ability of PPIn to act as signaling moieties involves the inositol headgroup. Indeed, it is the inositol headgroup that can be selectively phosphorylated by specific lipid kinases ( Figure 1A) at one of three positions (D-3, D-4, or D-5) to generate seven PPIn species from the parent, phosphatidylinositol (Ptdlns). Each of the seven PPIn species—three monophosphorylated phosphoinositides (PtdIns3 *P*, PtdIns4 *P*, and PtdIns5 *P*), three bisphosphorylated phosphoinositides (PtdIns(3,5) *P*_2_ [Phosphatidylinositol 3,5-bisphosphate], PtdIns(4,5) *P*_2_, and PtdIns(3,5) *P*_2_), and a single trisphosphorylated phosphoinositide (PtdIns(3,4,5) *P*_3 _[Phosphatidylinositol 3,4,5 trisphosphate]) ( Figure 1B)—has signature cellular locations ( Figure 2). For example, PtdIns4 *P* within the cell can be found at the plasma membrane (PM), endosomes, and trans-Golgi network, whereas the majority of PtdIns(4,5) *P*_2_ or PtdIns(3,4,5) *P*_3_ within cells are found mostly at the PM. Precise spatial regulation of PPIn distribution is critical for regulated cellular function and is carefully controlled through the catalytic actions of around 50 (34 phosphatases and 20 kinases) ^7^ differentially localized PPIn-metabolizing enzymes, each with highly specific preferences for a given PPIn species headgroup.

Despite contributing a small fraction to the bulk of cellular phospholipids, PPIn make striking contributions to practically every aspect of cell biology/physiology. They do so by recruiting and interacting with proteins at the membrane–cytoplasm interface to organize and shape organelle identity. There are many excellent reviews ^1, 8– 10^ that discuss PPIn distribution, metabolism, and function across many cell types; these articles are wonderful starting points to inform readers of the general principles and importance of these essential signaling lipids. This review article briefly summarizes our current understanding of the essential role(s) of PPIn in orchestrating and regulating crucial signaling events in the mammalian nervous system and puts particular emphasis on recent work. Further highlighting the roles of these lipids, we discuss the implications for human health and devastating disorders that arise when phosphoinositide metabolism goes awry.

## Biogenesis, distribution, and roles of polyphosphoinositides in the nervous system

To begin, we focus on the biogenesis of each individual PPIn species and their membrane distribution and define their cellular roles in healthy cells of the nervous system.

### Phosphatidylinositol

PtdIns, the precursor of all PPIn ( Figure 1A and B), is the most abundant PPIn species, contributing about 10 to 20 mol % of total membrane phospholipid content. The most abundant isoform is PtdIns 38:4, which (it should be noted) is different from the most abundant isoform in heterologous expression cell lines (PtdIns 36:1) ^11, 12^. The difference in fatty acid composition in cultured cells compared with primary cells remains to be fully determined. We know very little about its subcellular distribution despite being several orders of magnitude more concentrated in cellular membranes than other PPIn species. PtdIns is synthesized following the simple conjugation reaction of myo-inositol and CDP-DAG, catalyzed by a PtdIns synthase (PIS) enzyme in endoplasmic reticulum (ER) membranes. Following its biogenesis, PtdIns is transported out of the ER through one of the following three routes: (1) vesicular transport, (2) non-vesicular lipid transfer protein mechanisms at membrane contact sites ^13– 17^, or (3) via highly mobile PIS-containing vesicles ^18^. The use of lipid-binding domains for PtdIns4 *P* and PtdIns3 *P* has revealed that target membranes for PtdIns delivery include the plasma and Golgi membranes ^19– 21^ as well as a pool of endo-membranes ( Figure 1B). It remains to be seen whether significant amounts of PtdIns concentrate at these specific endo-membranes or it is rapidly transferred *de novo* to generate mono-phosphorylated species. Most investigations have focused on PtdIns as an essential precursor lipid for the generation of PtdIns4 *P* or PtdIns(4,5) *P*_2_^18^; this is almost certainly due to the lack of a faithful biosensor to rigorously investigate its distribution and metabolism.

For the nervous system, alterations in the concentration of one of the essential substrates for PtdIns synthesis, myo-inositol, or expressional change in a myo-inositol transporter, SMIT1 ( *SLC5A3* gene), modify neuronal excitability through downstream alterations in PtdIns(4,5) *P*_2_ metabolism ^22^ and direct interactions with KCNQ1/KCNE2 complexes ^23^, respectively. This information pairs well with older literature demonstrating that lithium, administered at therapeutically relevant doses, reduces myo-inositol and subsequently PtdIns to aid in the recovery of mood disorders, including bipolar affective disorder ^24, 25^. For PtdIns transfer proteins (PITPs), such as the Sec14-like or START-like proteins, there are strong links to human disease, such as the progressive neurodegenerative disorder vitamin E status ataxia with vitamin E deficiency (AVED) and a rare autosomal recessive disorder called Cayman-type cerebellar ataxia, to name a few (reviewed in 26). Further underscoring the importance of PITPs, a murine knockout model of PITPα presents striking neurological defects ^27^. Together, these data underscore the importance of PtdIns transport and metabolism for regulated nervous system function. Despite this knowledge, there are significant questions that remain unanswered in neurons, including the steady-state cellular distribution/metabolism of PtdIns and how this may be affected during signaling reactions or disease, and the role of membrane contact site proteins that transport PtdIns, such as TMEM24 ^13^. Hopefully, the development of tools to visualize PtdIns will offer helpful insights into some of these unanswered questions.

### Phosphatidylinositol 3-monophosphate

Phosphatidylinositol 3-monophosphate (PtdIns3 *P*) is the signature PPIn of endosomes and autophagosomes. Despite its relatively low abundance (20%–30% of PtdIns4 *P*), it is a key regulator of endocytic trafficking, fusion, and autophagy (for review, see 28) via PtdIns3 *P*-dependent interactions with PX or FYVE domains on proteins involved in cargo sorting, positioning, and maturation. PtdIns3 *P* is derived mainly from phosphorylation of PI by PI3K-II or PI3K-III ^29– 31^, and additional contributions are made from dephosphorylation of phosphatidylinositol 3,4-bisphosphate (PtdIns[3,4] *P*_2_) by PtdIns(3,4) *P*_2_ 4-phosphatases and PtdIns(3,5) *P*_2_ by PtdIns(3,5) *P*_2_ 5-phosphatases ( Figure 1B). For the nervous system, it has been reported that PI(3)P is involved (through WDR91–Rab7 interactions) in the regulation of dendritic arborization and post-natal development of the mouse brain ^32^, control of axonal transport and growth ^33^, and GABAergic neurotransmission at inhibitory post-synapses ^34^. Finally, underscoring a major role for PtdIns3 *P* in the nervous system, deletion of PIK3C3/Vps34 in sensory neurons causes rapid neurodegeneration ^35^.

### Phosphatidylinositol 4-monophosphate

Phosphatidylinositol 4-monophosphate (PtdIns4 *P*) can be directly synthesized from PtdIns at the plasma and Golgi membranes via the actions of PtdIns 4-kinases, with neuronal PM PtdIns4 *P* also potentially augmented via the actions of synaptojanins ^36^ and oculocerebrorenal syndrome of Lowe (OCRL) proteins ^37, 38^, which dephosphorylate PtdIns(4,5) *P*_2_ into PtdIns4 *P* ( Figure 1B). These two biosynthetic pathways, supplemented by PtdIns4 *P* generated by dephosphorylation of PtdIns(3,4) *P*_2_ by PtdIns 3-phosphatase enzymes, ensure that PtdIns4 *P* is found across several different organelle compartments, including the PM, Golgi, and endosomes ( Figure 2). All of the PtdIns 4-kinases ( Figure 1B) are expressed in the brain, and *PI4KA* (PI4KIIIα) and *PI4KB* (PI4KIIIβ) isoforms are localized throughout the nervous system. PI4KIIIα appears to be more highly expressed in spinal cord and cerebral cortex neurons, whereas PI4KIIIβ has enhanced distribution in the cerebellar cortex ^39, 40^. Localization studies from the Human Protein Atlas have revealed that *PI4K2A* (PI4KIIα) is expressed across different neuronal and astrocyte populations, and there are high levels in Purkinje cells, hippocampus, and dentate gyrus; *PI4K2B* (PI4KIIβ) is expressed in the cerebellum, and the highest expression is reported for the hippocampus. Taken together, there is a large body of evidence that each of these enzymes is localized throughout the brain, including in many classes of neuron.

In the peripheral nervous system, PI4KIIIα was recently reported to play an essential role in myelin formation as Schwann cell–specific inactivation of the gene caused myelination defects and gross alterations in actin architecture ^41^. Currently, there is little direct information visualizing the distribution of PtdIns4 *P* in central nervous system neurons. Information gained from sympathetic superior cervical ganglia (SCG) neurons ^11^, expressing a biosensor for PtdIns4 *P* (P4M) ^42^, suggests that a significant portion of the lipid resides at the PM at rest and that other pools are in intracellular organelles (likely the trans Golgi and endosomes). Such a distribution is consistent with other reports from mammalian expression system cells ^42, 43^, suggesting a conserved localization of PtdIns4 *P*-metabolizing enzymes. Interestingly, the same authors [11] revealed a threefold accelerated synthesis of PM PtdIns4 *P* in SCG neurons, suggesting higher enzymatic activity of the lipid 4-kinase. Thus, there may be subtle differences in enzyme abundance, activity, and localization in primary neuronal cells. Refined experimental designs/tools will be necessary to analyze the molecular mechanisms underlying the accelerated synthesis of PM PtdIns4 *P* in neurons. For the other main cellular source of PtdIns4 *P*, the trans Golgi, information from non-neuronal cells reveals that *PI4KB* and *PI4K2A* and -2B all contribute to its synthesis. *PI4KB* is recruited to the Golgi by Arf1 ^44– 46^, whereas *PI4K2A* and -2B contribute to Golgi PtdIns4 *P* via lipid modifications and perhaps cholesterol-rich domains ^47– 51^.

Both PM and Golgi PtdIns4 *P* pools appear under further regulatory control by the lipid transfer proteins ORP5/8 (oxysterol-binding protein-related proteins5/8) ^52– 55^ and OSBP (oxysterol-binding protein) ^56, 57^, respectively. At the PM, ORP5/8 are localized to regions of close proximity (15–20 nm) between the ER and PM, termed ER-PM contact sites. These membrane contacts visualized in excitable cells ^58– 60^, including neurons ^61^, are sites of close organelle membrane apposition that facilitate information transfer (lipids and ions), independent of vesicular transport. Such membrane fusion-independent lipid transport is likely to be essential in complex cells, like neurons, where organelle compartments are often separated by large distances. Through binding of their N-terminal pleckstrin homology (PH) domains with PtdIns4 *P* ^55^ or PtdIns(4,5) *P*_2_^53^ or both ^52^, the ER-localized ORP5/8 dock with the PM. Despite not being functionally characterized in neurons, the ubiquitously expressed ORP5/8, similar to other mammalian cells, are likely to facilitate the counter-transport of phosphatidylserine (to the PM) for PtdIns4 *P* (to the ER). Transported PtdIns4 *P* is then likely to be dephosphorylated to PtdIns by the ER PtdIns4 *P*-4-phosphatase, Sac1. Thus, ORP5/8 may serve not only to tune PM PtdIns4 *P* but also to aid in the maintenance of ER PtdIns levels. At ER–Golgi membrane contact sites, OSBP1 also serves to regulate PtdIns4 *P* abundance. Once positioned at ER–Golgi membrane contact sites, OSBP exchanges cholesterol (on ER membrane) for PtdIns4 *P* (on trans-Golgi membrane) ^56, 57^. Compelling evidence for the importance of PtdIns4 *P* in the nervous system is demonstrated by *PI4K2A* gene-trapped mice developing late-onset spinocerebellar axonal degeneration and the presence of *PI4K2A* on synaptic vesicles ^62^.

### Phosphatidylinositol 5-monophosphate

Phosphatidylinositol 5-monophosphate (PtdIns5 *P*) remains the most enigmatic of the PPIs because of its low abundance (similar to that of PtdIns3 *P*) and the current lack of a faithful biosensor. It is for these reasons that the effectors controlled by PtdIns5 *P* and the pathways it regulates are poorly understood relative to the other PPIn family members. How PtdIns5 *P* is biosynthesized remains controversial. Work on non-neuronal mammalian cells suggests two pathways for its generation: (1) directly by phosphorylation of PtdIns by a PI 5-kinase (such as PIKfyve or type I PI5K enzymes) ^63– 65^ or (2) indirectly via dephosphorylation of PtdIns(3,5) *P*_2_ by the myotubularin phosphatases ^66^. PtdIns5 *P* was initially discovered as having a signaling role in the nucleus ^67, 68^ since reports of PtdIns5 *P* being involved in Akt/mammalian target of rapamycin (Akt/mTOR) signaling ^69^ and apoptosis ^70^ have been documented (for review see 71, 72. For the nervous system, there is little direct information regarding PtdIns5 *P*.

### Phosphatidylinositol 4,5-bisphosphate

Phosphatidylinositol 4,5-bisphosphate (PtdIns[4,5] *P*_2_) is the signature PPIn of the PM ( Figure 2) and undoubtedly the best-characterized PPIn of the nervous system. It is produced primarily through the phosphorylation of PtdIns4 *P* by type I PtdIns4 *P* 5-kinases (α, β, and γ), although there may be minor contributions from PtdIns(3,4,5) *P*_3_ 5-phosphatases (PTEN) or PtdIns5 *P* 4-kinases ( Figure 1B). PtdIns(4,5) *P*_2_ is under a further layer of regulation from PtdIns(4,5) *P*_2_ 5-phosphatases, like synaptojanin 1 and 2 and OCRL (Figures 1B and 2). Underscoring the importance of these enzymes for human health, mutations in the genes that encode the PtdIns(4,5) *P*_2_ 5-phosphatases result in a host of human disorders of the nervous system, including seizures ^73^, Alzheimer’s ^74^, Down syndrome ^75^, Parkinson’s ^76^, and Lowe syndrome ^37^.

**Figure 3.**
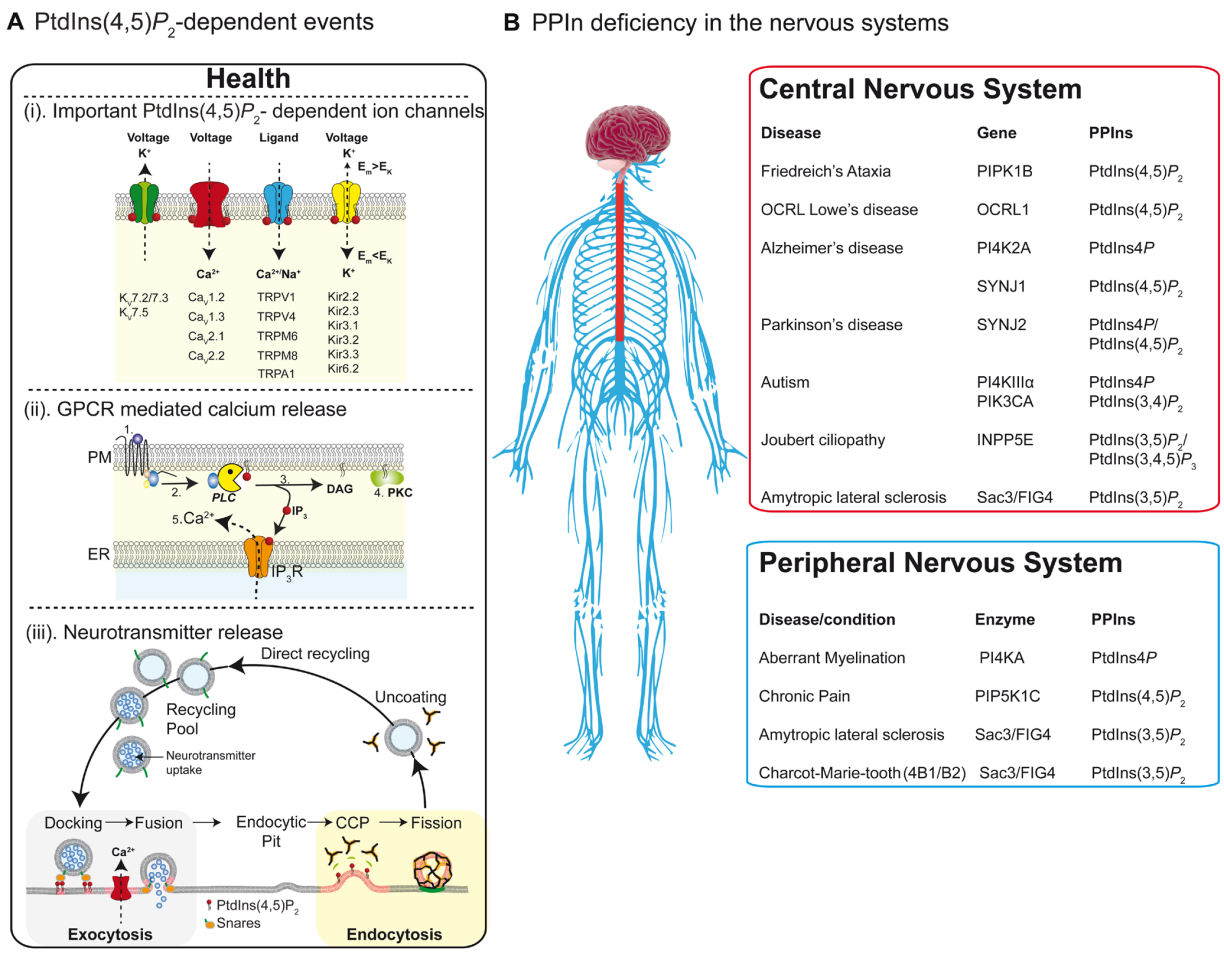
Roles for polyphosphoinositides (PPIn) in the nervous system in health and disease. ( **A**) PtdIns(4,5) *P*_2_-dependent events. Critical events regulated by plasma membrane (PM) PtdIns(4,5) *P*_2 _within the nervous system. (i) Four families of ion channels that require PtdIns(4,5) *P*_2_ as a co-factor for full function. (ii) PtdIns(4,5) *P*_2_ is the critical precursor for generation of IP _3_-mediated Ca ^2+^ release and protein kinase C (PKC)-mediated phosphorylation. Binding of ligand (1) releases the heterotrimeric G-protein G _q_ (2) to activate phospholipase C (PLC), which subsequently hydrolyses PM PtdIns(4,5) *P*_2_ into membrane-bound DAG and soluble IP _3_ (3). DAG then can recruit PKC to phosphorylate protein targets (4) while IP _3_ binds to the IP _3_R on endoplasmic reticulum (ER) membranes to initiate Ca ^2+^ releases into the cytoplasm (5). (iii) Critical involvement of PtdIns(4,5) *P*_2_ in neurotransmitter release. During calcium-regulated synaptic vesicle release, PtdIns(4,5) *P*_2_ is required to attract many proteins to the PM active zone for docking and fusion. After fusion, the vesicle membrane is recovered via the clathrin adapter protein AP2 to form clathrin-coated pits (CCP), before dynamin-dependent membrane scission occurs during the final stages of endocytosis. ( **B**) PPIn deficiency in the nervous system. Diseases and cellular consequences for altered PPIn metabolism in the central (red box) and peripheral (blue box) nervous systems. CCP, clathrin coated pit; GPCR, G protein–coupled receptor; PtdIns, phosphatidylinositol.

PtdIns(4,5) *P*_2_ plays an essential role in regulating many essential PM events, including electrical signaling ( Figure 3Ai), synaptic plasticity ^77^, endocytosis, and exocytosis ( Figure 3Aiii). It also acts as a substrate for phospholipase C (PLC) following G protein–coupled receptor (GPCR) activation ( Figure 3Aii). To date, around 100 ion channels and transporters have been shown to be directly regulated by this lipid (for review, see [10]); many of these PtdIns(4,5) *P*_2_-sensitive channels, including voltage-gated potassium channels ^78– 80^ and voltage-gated calcium channels ^81^, are found in cells of the nervous system ( Figure 3Ai). Thus, alterations in abundance or distribution (or both) of this minor lipid can significantly alter electrical activity in neurons ^11, 82^. One such mechanism that dynamically modulates PM PtdIns(4,5) *P*_2_ abundance is binding of modulatory neurotransmitters to receptors coupled to PLC ( Figure 3ii). The consequence of PLC activation is rapid hydrolysis of PtdIns(4,5) *P*_2_ into soluble IP _3_ and membrane-bound diacylglycerol (DAG). Consequently, modulatory neurotransmitters of the nervous system that couple to G _q_ have the potential to nearly synchronously switch off specific ion channels, initiate Ca ^2+^ from IP _3_R on ER membranes, and recruit protein kinase C (PKC) to the PM. Termination of these signaling reactions, following removal of neurotransmitter from the synaptic cleft, allows PtdIns(4,5) *P*_2_ to be rapidly resynthesized ^11^. The source or sources of PtdIns4 *P* that serve as precursor sources for the PM PtdIns(4,5) *P*_2_ pool that supports ion channel activity appear to originate from the PM ^11, 83^ and trans-Golgi membranes ^84^.

### Phosphatidylinositol 3,4-bisphosphate

PtdIns(3,4) *P*_2_ is localized mainly to the PM and endocytic compartments ( Figure 2), where it typically interacts with TAPP domain-containing proteins to orchestrate signaling cascades. Current evidence suggests that the majority of PtdIns(3,4) *P*_2_ is formed through the actions of INPP5D (SHIP1) and INPPL1 (SHIP2) phosphatases following class I PI3K-mediated generation of PtdIns(3,4,5) *P*_3_ at the PM ^85– 87^ ( Figure 1B and 2) or by phosphorylation of PtdIns4 *P* via class II PI3K lipid kinases ^88^. PtdIns(3,4) *P*_2_ is under a further layer of regulation through the metabolic phosphatase actions of INPP4A/B ^89^ and PTEN ^87^, which act on their substrates to generate PtdIns3 *P* and PtdIns4 *P*, respectively. PtdIns(3,4) *P*_2_ is involved in maturation of late-stage clathrin-coated pits [89] and in fast endophilin-mediated endocytosis ^90, 91^. Loss of INPP4A function leads to neurodegeneration through a mechanism thought to involve enhanced neuronal susceptibility to glutamate-induced excitotoxicity ^89^, underscoring a potential neuroprotective role for INPP4A by tuning PtdIns(3,4) *P*_2_ signaling pathways. Finally, clustering of PtdIns(3,4) *P*_2_ appears necessary and sufficient for actin-mediated neurite initiation and dendrite morphogenesis ^92^. Thus, PtdIns(3,4) *P*_2_ is thought to play a role in the maintenance of nervous system function via its role at the PM, endocytotic membranes, and more distal membrane compartments.

### Phosphatidylinositol 3,5-bisphosphate

PtdIns(3,5) *P*_2_ is the low-abundance (<0.1% of total PPIs) signature PPIn of late endosomal/lysosomal membranes ( Figure 2). Current knowledge indicates that the synthesis and turnover of PtdIns(3,5) *P*_2_ are tightly controlled by a large protein complex that includes Vac14, PIKfyve, and FIG4. Through direct interactions, Vac14 nucleates the complex between the PtdIns3 *P* 5-kinase (PIKfyve) and PtdIns(3,5) *P*_2_ 5-phosphatase (Sac3/FIG4) to ensure tight coordination between synthesis and degradation of PtdIns(3,5) *P*_2_^66, 93^ ( Figure 1B). At steady state, the relatively low abundance of PtdIns(3,5) *P*_2_ is important for membrane trafficking, endocytic vesicle fission/fusion, organelle pH, and intracellular ion channel function ^94– 98^. Mouse models of Fig4 and Vac14 deletions and a mutation within PIKfyve exhibit embryonic lethality or severe neurodegenerative phenotypes ^66, 99, 100^. PtdIns(3,5) *P*_2_ abundance has also been correlated with long-term depression, and the activity of PIKfyve is seemingly involved in modifying synaptic strength ^100, 101^. Together, these observations suggest that PtdIns(3,5) *P*_2_ is essential for proper development in the nervous system.

### Phosphatidylinositol 3,4,5 trisphosphate

PtdIns(3,4,5) *P*_3_ is generated following binding of extracellular stimuli—for example, growth factors epidermal growth factor (EGF), platelet-derived growth factor (PDGF), and insulin-like growth factor-I (IGF-I)—to receptors that activate class I PI3K to phosphorylate PtdIns(4,5) *P*_2_ into PtdIns(3,4,5) *P*_3_ ( Figure 1B). Receptor-mediated elevations in PtdIns(3,4,5) *P*_3_ lead to recruitment of protein kinases (for example, AKT, BKT, and PDK1) to the PM to shape downstream cellular signaling cascades. PtdIns(3,4,5) *P*_3_/PI3K activity has been implicated in many facets of nervous system function; for example, PtdIns(3,4,5) *P*_3_ appears to be involved in clustering Syntaxin1A to regulate neurotransmitter release ^102^, while levels of Akt regulate axon branching, formation of dendritic spines, cell hypertrophy, growth cone expansion, and axon regeneration in neurons ^103– 105^. PtdIns(3,4,5) *P*_3_ levels are under tight regulatory control by the catalytic activity of the protein and lipid phosphatase, PTEN. PTEN is widely expressed in mouse brain, and there is some preferential distribution in Purkinje neurons and some pyramidal neurons ^106^, where it is thought to be involved in neuronal migration, size, and survival ^106, 107^. Interestingly, PTEN has been reported as a potential target for neuroprotection and neuroregeneration following insult or injury (for review, see 108). In these studies, upregulation of mTOR activity in corticospinal neurons via conditional deletion of PTEN, a negative regulator of mTOR, enables successful regeneration of a group of injured axons ^109^. Along similar lines, conditional inactivation ^110^ or inhibition ^111^ of PTEN function in oligodendrocytes is required to regulate myelin thickness and preserve axon integrity.

## Polyphosphoinositides in diseases of the nervous system

In health, the PPIn zip code ( Figure 2) is established through the combined spatial and temporal activities of over 50 PPI-metabolizing enzymes [7]. Through the actions of each of the 34 PPIn phosphatases and 20 PPIn kinases, each of the seven PPIn species is generated and interacts with over 400 different proteins at the membrane–cytosol interface ^112^. Given the sheer coverage of the intracellular PPIn interactome, it is perhaps not surprising that mutations in phosphoinositide kinases and phosphatases have been implicated in many human diseases of the nervous system ( Figure 3B). To date, over 20 monogenetic disorders have been reported to be caused by mutations in PPIn enzymes. Indeed, the role of PPIn phosphatases and kinases in health and disease has been covered comprehensively in several reviews ^7, 113– 116^. Here, we focus our attention on a few of the more commonly occurring neurological disorders that have been suggested to arise through defects in PPIn metabolism.

Several studies have suggested that mutations in genes coding for PPIn-metabolizing enzymes are associated with autism spectrum disorders. Interestingly, the majority of the PPIn enzymes associated with autism are PPIn kinases, and isoforms of the class 1 PI3K family (for review, see 117), PI4K ^118^, and PIP5K ^119^ are all reported to play prominent roles. For the PI4Ks, mutations in the peripheral membrane adaptor protein of the PI4KIIIα signaling complex, EFR3, are significantly more common among autism spectrum cases than controls ^118^. Thus, considerable evidence suggests that involvement in PPIn signaling in autism; however, how each of these PPI-metabolizing proteins contributes to the pathophysiology awaits further delineation.

Amyotrophic lateral sclerosis (ALS), commonly known as Lou Gehrig’s disease, is a progressive neurodegenerative disease characterized by selective motor neuron death leading to muscle atrophy, paralysis, and motor impairment. Currently, two proteins related to PPIn metabolism have been determined to be disease-causing ALS mutations. The first is a substitution of proline with serine at residue 56 on the vesicle-associated membrane protein (VAMP)-associated protein (VAP) VAPB gene (P56S; designated ALS8). VAPB is a conserved integral membrane protein of the ER found in all eukaryotic cells and regulates PPIn transport and homeostasis at ER-membrane contact sites ^14, 15, 120– 122^. At present, the molecular mechanism or mechanisms underlying ALS8 pathogenesis remain poorly understood; however, in transgenic mice, expression of human VAPB with the ALS8 mutation causes various motor behavioral abnormalities, including progressive hyperactivity ^123^. Thus, future investigations appear warranted to determine the downstream neuropathology associated with this mutation. The second PPIn protein associated with ALS involves the PtdIns(3,5) *P*_2_ 5-phosphatase, Sac3/FIG4. Mutations in Sac3/Fig4 result in a significant loss of protein function, resulting in this autosomal dominant form of ALS, designated ALS11 [99]. Further implicating alterations in PtdIns(3,5) *P*_2_ metabolism as a potential risk factor for disease progression, mutations in PIKfyve production have been linked to neurological disorders such as ALS and Charcot–Marie–Tooth disease ^66, 124^. Indeed, mutations in myotubularin-related 2 (MTMR2), which preferentially dephosphorylates PtdIns3 *P* and PtdIns(3,5) *P*_2_ into PtdIns and PtdIns5 *P*, respectively, cause autosomal recessive Charcot–Marie–Tooth disease type 4B (CMT4B1) ^125– 127^. This disorder manifests as childhood onset of progressive muscle weakness of the distal muscles and sensory loss that is characterized by decreased nerve conduction velocity and demyelination in the nerve ^125^. A less severe MTMR2 ^−/−^ mouse model develops azoospermia and abnormal peripheral nerve myelination with marked myelin sheath focal outfoldings in Schwann cells rather than peripheral motor neurons.

A growing body of evidence suggests that intracellular levels of PPIn are significantly altered in the two most prevalent neurodegenerative disorders: Alzheimer’s ^74, 128, 129^ and Parkinson’s ^73, 76, 130^. For Alzheimer’s disease, genetic polymorphisms or mutations in genes such as INPP5D ^131^ and SYNJ1 ^132^ are risk factors for late-onset Alzheimer’s disease (LOAD), and there are several reports of amyloid beta–dependent alterations in the catalytic activity of synaptojanin ^133^ and PI4K2A ^134^. For Parkinson’s disease, an autosomal recessive R258Q mutation within the Sac domain of synaptojanin 1 was recently designated PARK20 ^76^. At the cellular level, this mutation alters synaptic development and this is accompanied by endocytic defects and accumulation of clathrin-coated intermediates. At the behavioral level, mice harboring this mutation develop neurological symptoms similar to those of human patients. Further emphasizing the link between dysfunction in early endocytic traffic and Parkinson’s disease, loss-of-function mutations in the ER-lysosome tethering protein VPS13C result in a distinct form of early-onset parkinsonism characterized by rapid and severe disease progression and early cognitive decline ^135, 136^.

These highlighted examples fully underscore the importance of regulated PPIn metabolism for human health. Given the ubiquitous distribution of PPIn across all mammalian cells, the scale of the PPIn interactome, and their essential role in choreographing critical signaling events, it is perhaps inevitable that every human disease will exhibit some form of PPIn dysfunction.

## Conclusions and future directions

In the past 20 years, there has been an explosion of research on PPIn signaling. The overarching narrative of this work is that PPIn are indispensable and universal signaling entities that initialize, organize, and contribute to nearly all aspects of cellular life. Despite these heroic efforts, there is a lack of information that translates and integrates what we understand in expression systems to crucial primary cells like neurons. This author is especially excited to better understand the neuronal localization and function of each of the enzymes listed in Figure 1B. For membrane contact sites, very little is known in neurons apart from beautiful characterizations of their morphology. Simple questions remain unanswered, such as their primary roles, the consequence(s) of their absence, and heterogeneity/redundancy of proteins within defined membrane contact sites. For disease, we need to determine at the molecular level how alterations in PPIn at specific organelle membranes translate to progressive changes in human behavior, ultimately leading to neuropathies and frequently death. Finally, with continued development of pharmacological tools, investigators can begin leveraging what we know about PPIn metabolism (and their broad control of cellular reactions across multiple membranes) to potentially relieve symptoms of disease without actually addressing the underlying genetic or idiopathic factors initiating the disease.

In conclusion, PPIn play a central role in coordinating virtually all aspects of a cell’s life and death. Such fundamental involvement demands continued research into the biology of PPIn, specifically primary cells (like neurons), with the goal to develop diagnostics and novel therapeutic strategies to expedite treatment of human disorders.

## Abbreviations

ALS, amyotrophic lateral sclerosis; ER, endoplasmic reticulum; mTOR, mammalian target of rapamycin; OCRL, oculocerebrorenal syndrome of Lowe; ORP, oxysterol-binding protein-related protein; OSBP, oxysterol-binding protein; PtdIns, phosphatidylinositol; PI, phosphoinositide; PIS, phosphatidylinositol synthase; PITP, polyphosphoinositide transfer protein; PLC, phospholipase C; PM, plasma membrane; PPIn, polyphosphoinositides; PTEN, phosphatase and tensin homolog; SCG, superior cervical ganglia; VAP, vesicle-associated membrane protein-associated protein
